# Supplementary figures and images for: Microbiome patterns across the gastrointestinal tract of the rabbitfish Siganus fuscescens
Source: PeerJ. 2017 May 17;5:e3317. doi: 10.7717/peerj.3317 (PMC5437856; doi:10.7717/peerj.3317)

**a)**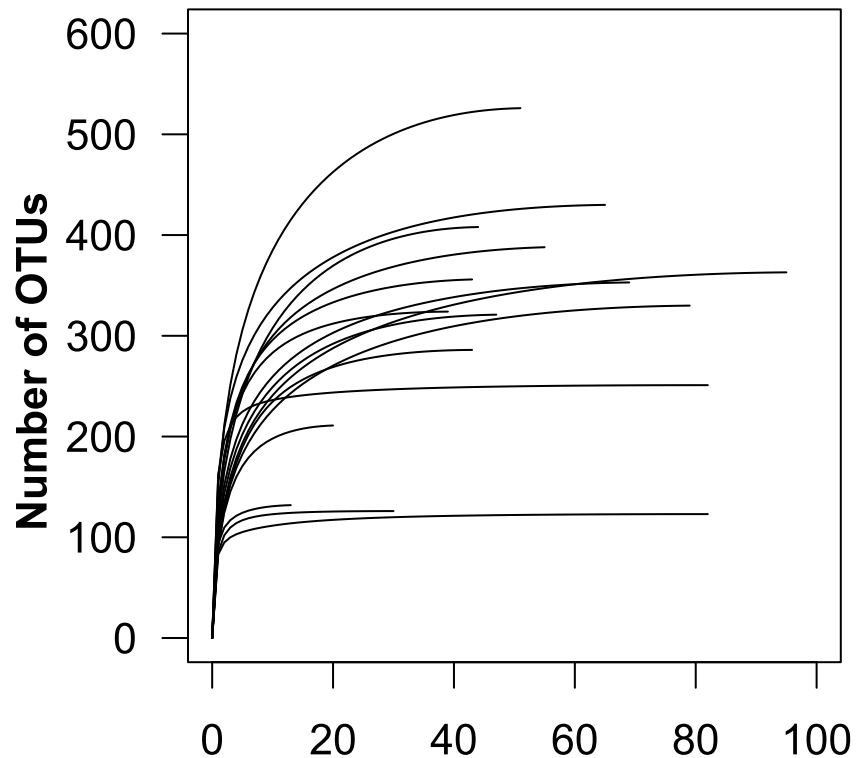**b)**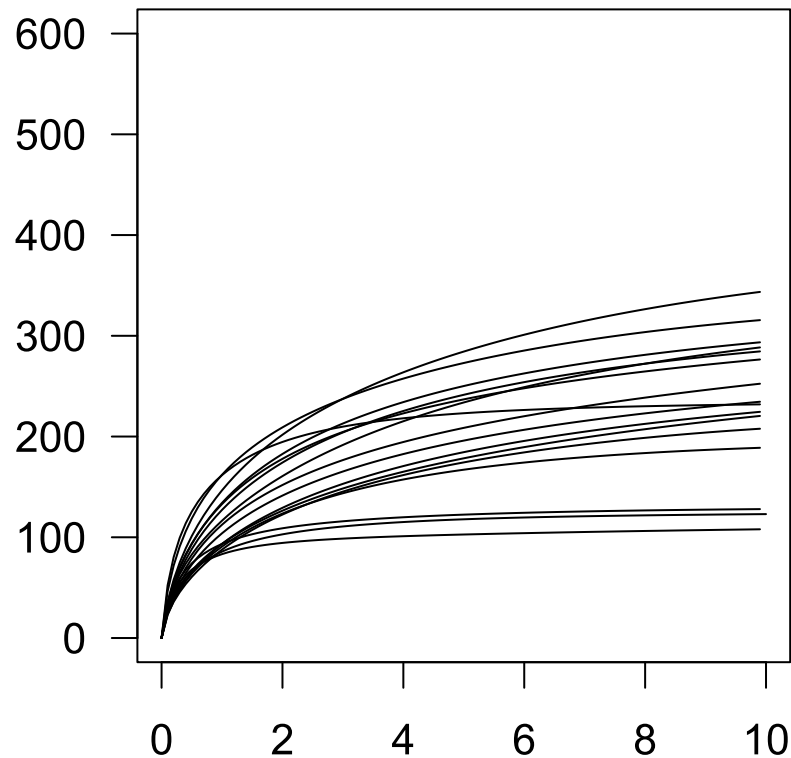**Sequences samples (x 1000)**

Supplement: Figure S1 — Rarefaction curves of microbial communities sampled from the gut of mottled spinefoot rabbitfish (Siganus fuscescens), a) after sequence quality filtering and with differential sequencing depths, and b) equalized sampling depths (10,000 sequences randomly obtained per sample). [file peerj-05-3317-s001.pdf]

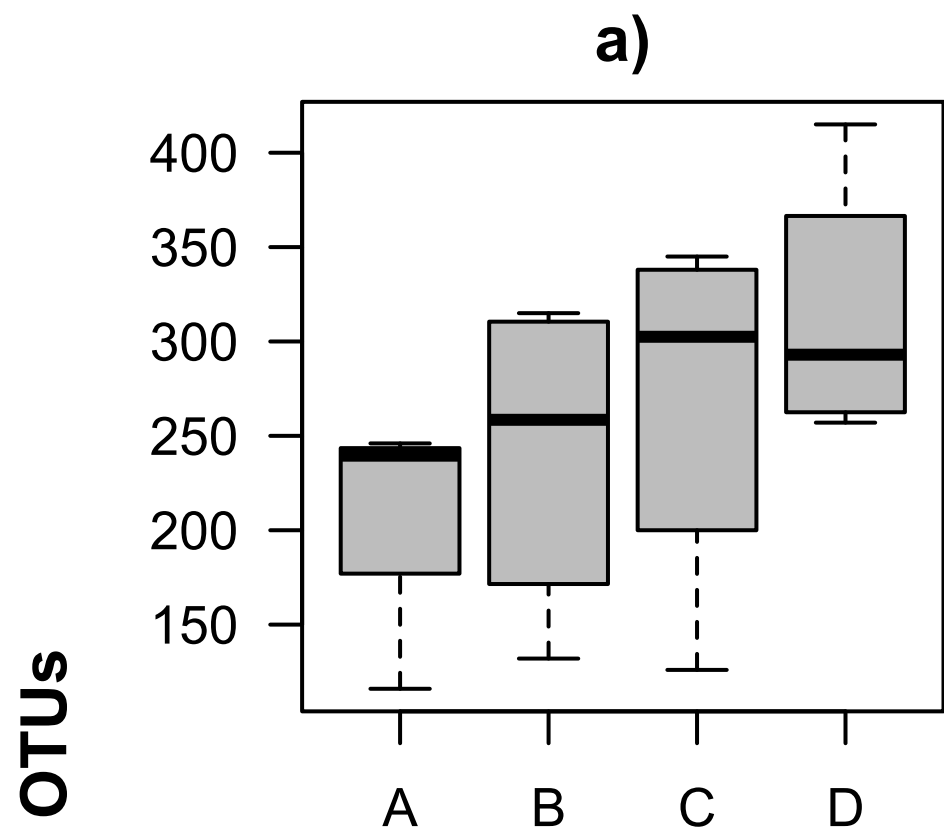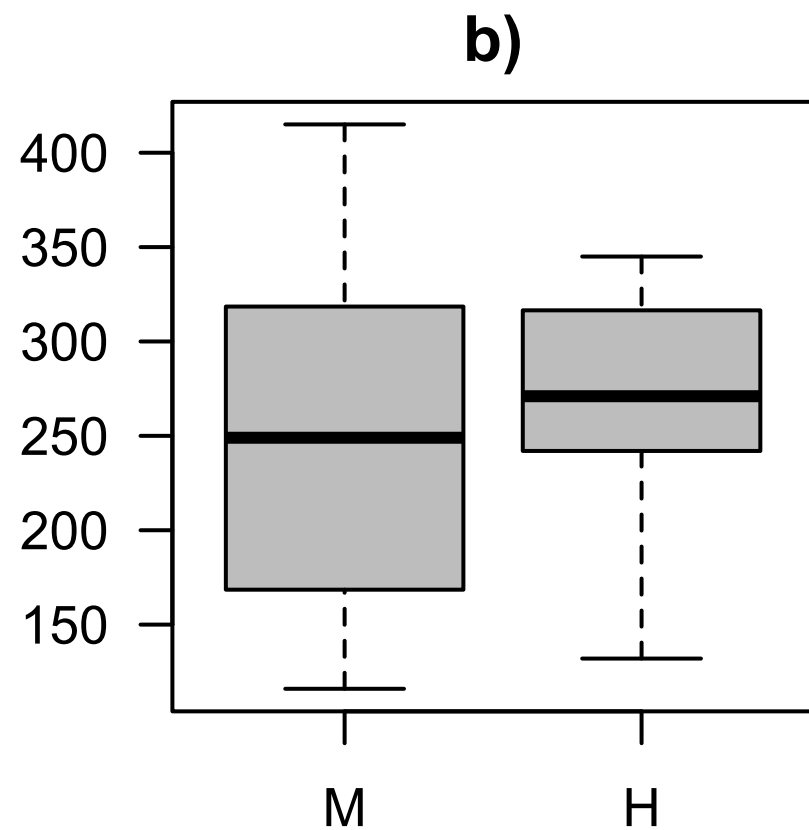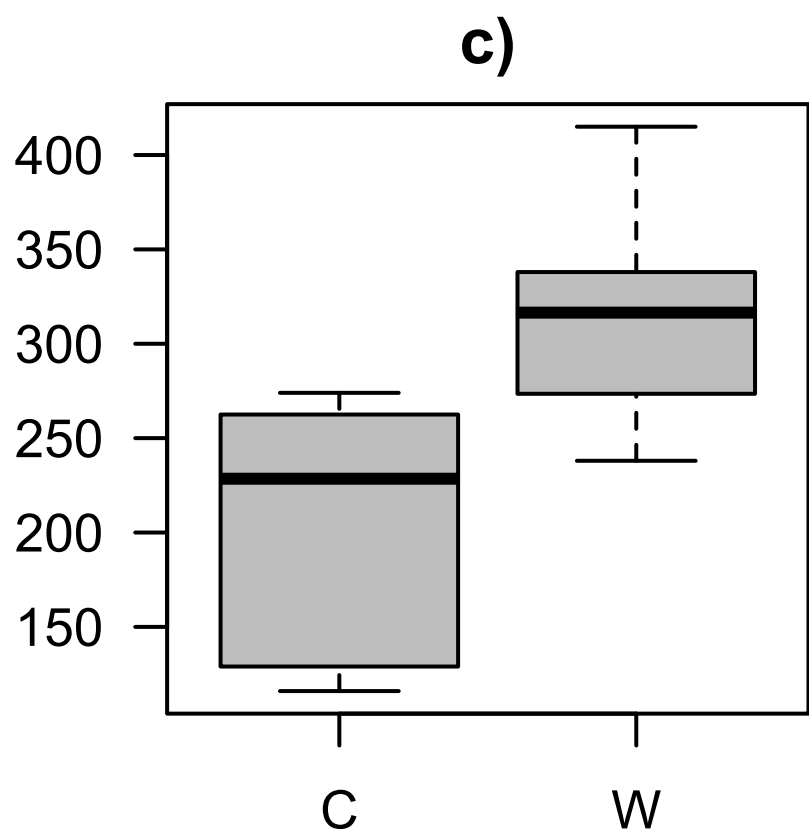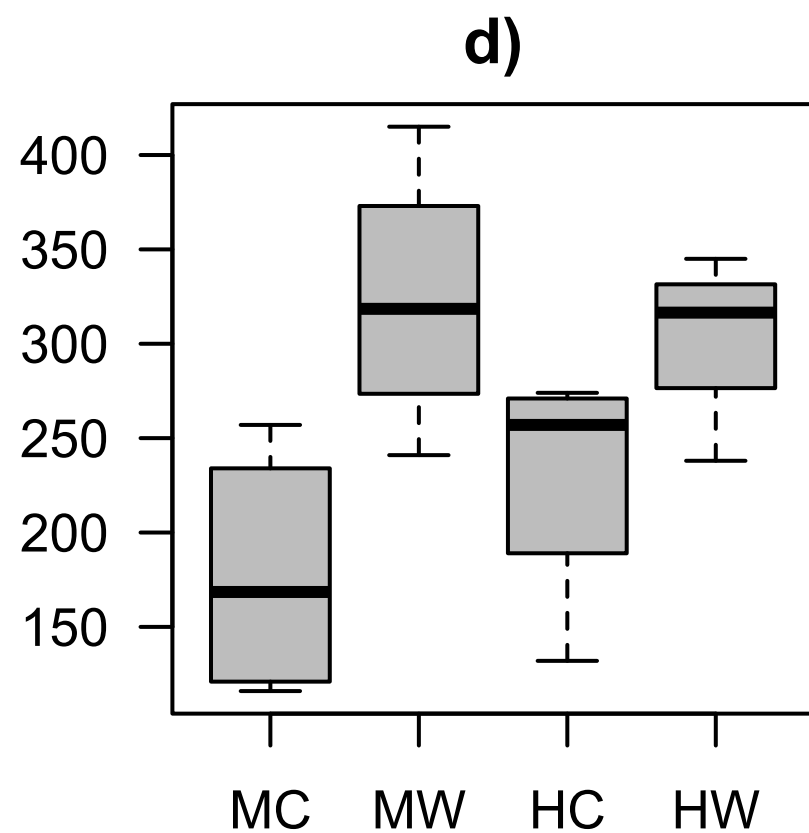

**Sample group**

Supplement: Figure S2 — Bacterial species richness (number of OTUs) within the gut of the mottled spinefoot rabbitfish (Siganus fuscescens) compared among a) individual fish (n = 4), b) gut locations (M = mid, H = hind), c) gut sites (C = content, W = wall) and d) gut sites within gut locations (MC = mid-content, MW = mid-wall, HC = hind-content, HW = hind-wall), given a total sampling depth of 10,000 16S rRNA gene counts per sample [file peerj-05-3317-s002.pdf]

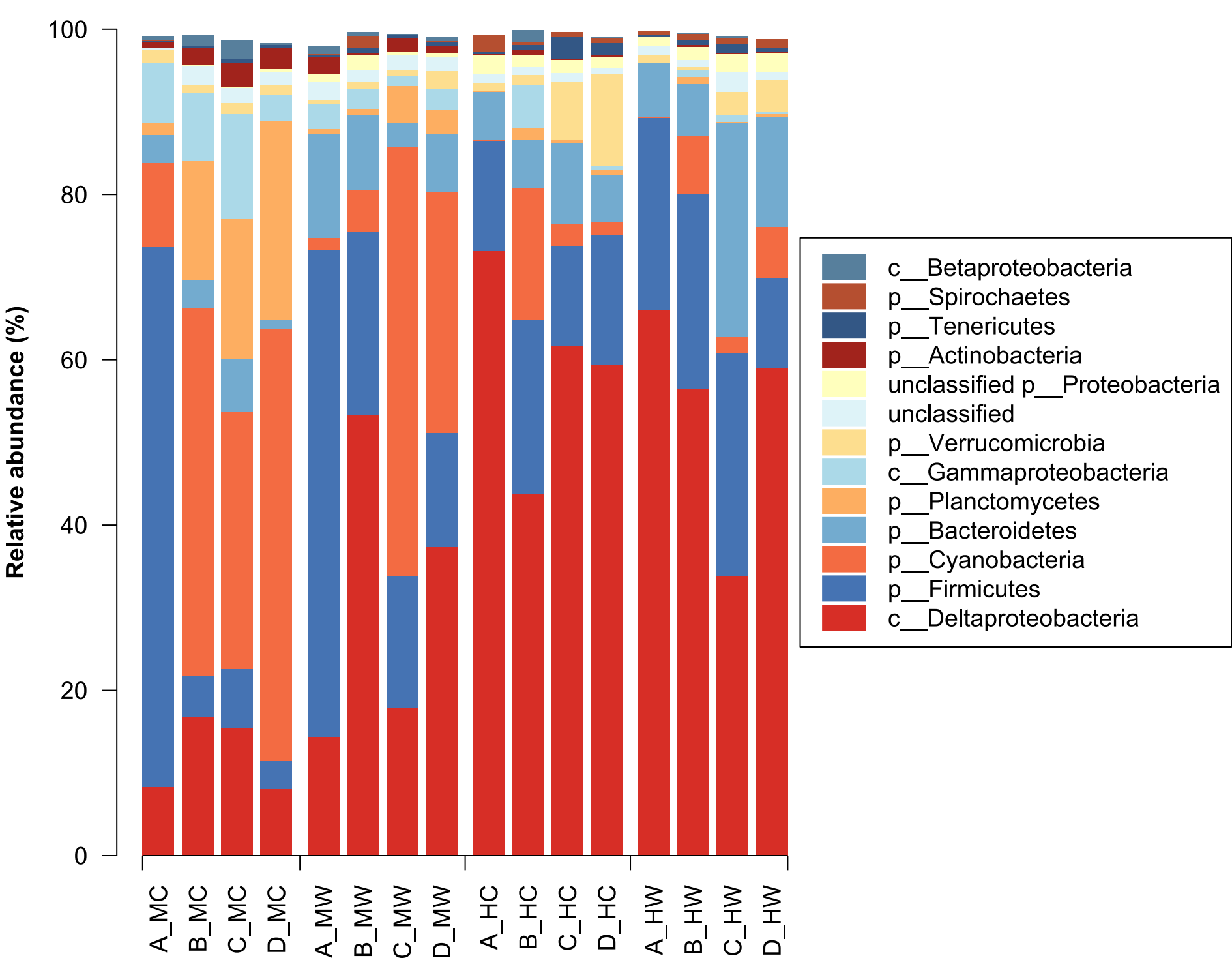

Supplement: Figure S3 — Only phyla with relative abundances >1% are shown, and the phylum proteobacteria has been split into it’s component classes. Horizontal axis labels represent Fish individual (A–D) followed by an underscore and then gut sites within gut locations (MC = mid-content, MW = mid-wall, HC = hind-content, HW = hind-wall). [file peerj-05-3317-s003.pdf]

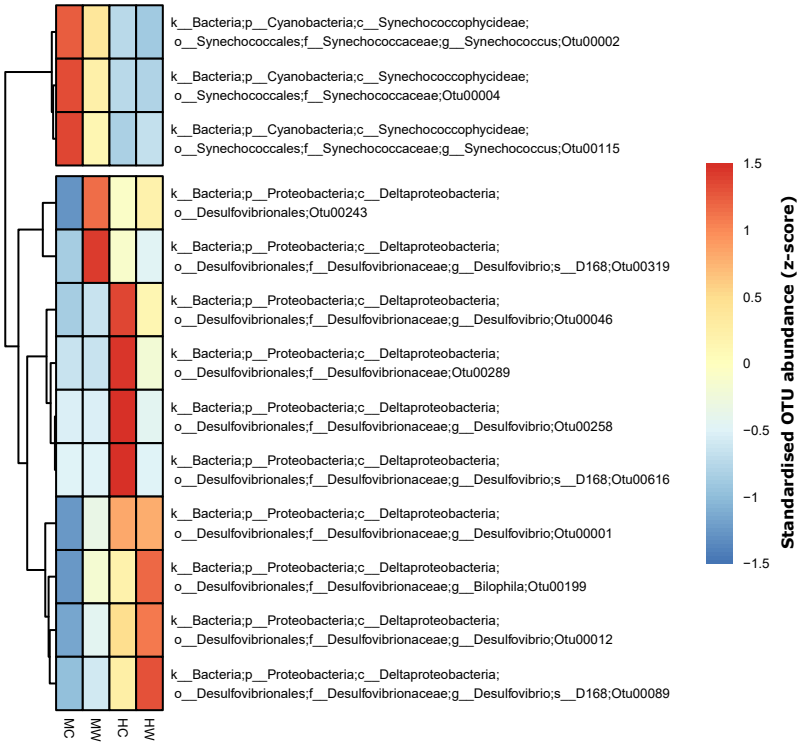

Supplement: Figure S4 — Standardised abundances (z-score transformation) of OTUs associated with the taxonomic groups o_Desulfovibrionales and f_Synechococcaceae among gut sites within gut locations (MC = mid-content, MW = mid-wall, HC = hind-content, HW = hind-wall) within the gut of the mottled spinefoot rabbitfish (Siganus fuscescens). Only OTUs with relative abundances >0.01% are shown. [file peerj-05-3317-s004.pdf]
