# Supplementary material for: Microbiome patterns across the gastrointestinal tract of the rabbitfish Siganus fuscescens
Source: PeerJ. 2017 May 17;5:e3317. doi: 10.7717/peerj.3317 (PMC5437856; doi:10.7717/peerj.3317)
Supplement: Table S1 — ANOVA of a) species richness and b) species diversity, and c) PERMANOVA of species composition among Fish, Gut locations and Sites within the gut of the mottled spinefoot rabbitfish (Siganus fuscescens). SD = Standard deviation among fixed effect estimates (a proxy for the random effect variation among Fish). [file peerj-05-3317-s005.pdf]

Table S1. ANOVA of a) species richness and b) species diversity, and c) PERMANOVA of species composition among Fish, Gut locations and Sites within the gut of the mottled spinefoot rabbitfish (*Siganus fuscescens*). SD = Standard deviation among fixed effect estimates (a proxy for the random effect variation among Fish).

a) ANOVA of species richness (number of OTUs observed), see Figure S3.

**Source**	**Df**	**MS**	**F value**	**P**	**SD**
Fish	3	7645	2.82	0.100	43.72
GutLoc	1	1073	0.40	0.545	
Site	1	50288	18.54	0.002	
GutLoc:Site	1	6848	2.52	0.147	
Residuals	9	2712			

b) ANOVA of Species diversity (Shannon-Weaver diversity index), see Figure 1.

**Source**	**Df**	**MS**	**F value**	**P**	**SD**
Fish	3	0.08	0.23	0.873	0.14
GutLoc	1	0.55	1.64	0.232	
Site	1	0.64	1.91	0.200	
GutLoc:Site	1	0.03	0.10	0.759	
Residuals	9	0.33			

c) PERMANOVA of Bray-Curtis dissimilarities, see Figure 2.

**Source**	**Df**	**MS**	**F value**	**P**
Fish	3	0.22	2.24	0.009
GutLoc	1	0.55	5.67	0.001
Site	1	0.26	2.72	0.011
GutLoc:Site	1	0.14	1.48	0.133
Residuals	9	0.10		
